# Antioxidative and Anticholinesterase Activity of *Cyphomandra betacea* Fruit

**DOI:** 10.1155/2013/278071

**Published:** 2013-11-03

**Authors:** Siti Hawa Ali Hassan, Mohd Fadzelly Abu Bakar

**Affiliations:** Institute for Tropical Biology and Conservation, Universiti Malaysia Sabah (UMS), Jalan UMS, 88400 Kota Kinabalu, Sabah, Malaysia

## Abstract

*Cyphomandra betacea* is one of the underutilized fruits which can be found in tropical and subtropical countries. This study was conducted to determine the antioxidant activity and phytochemical contents in different parts (i.e., flesh and peel) of the fruits. Antioxidants were analyzed using DPPH and ABTS free radical scavenging assays as well as FRAP assay. Anticholinesterase activity was determined using enzymatic assay using acetyl cholinesterase enzyme. For 80% methanol extract, the peel of the fruit displayed higher antioxidant activity in both FRAP and ABTS free radical scavenging assays while the flesh displayed higher antioxidant activity in the DPPH assay. Total phenolic and total flavonoid content were higher in the peel with the values of 4.89 ± 0.04 mg gallic acid equivalent (GAE)/g and 3.36 ± 0.01 mg rutin equivalent (RU)/g, respectively. Total anthocyanin and carotenoid content were higher in the flesh of the fruit with the values of 4.15 ± 0.04 mg/100 g and 25.13 ± 0.35 mg/100 g. The anticholinesterase was also higher in the peel of *C. betacea*. The same trends of phytochemicals, antioxidant, and anticholinesterase were also observed in the distilled water extracts. These findings suggested that *C. betacea* has a potential as natural antioxidant-rich nutraceutical products.

## 1. Introduction

Coronary heart diseases, cancer, and other health problems related to the aging process have been associated with free radicals such as reactive oxygen species (ROS) [1]. The oxidation of the cell by ROS contributes to the cell degeneration which causes some chronic diseases such as cancer and Alzheimer's disease. However, the oxidation process by ROS can be reduced by antioxidants. Natural and synthetic antioxidants have been added to food and function to extend the shelf life of the products. In addition to that, the most important function of antioxidants in the human body is to combat ROS which help to control oxidation of cells in the human body [2]. Synthetic antioxidants have been shown to possess carcinogenic activity [3], hence natural antioxidants which are shown to be safer and have been comprehensively shown by researchers to possess antiviral, antimutagenic, antiinflammatory, anticancer, antitumor, and hepatoprotective properties [2]. 

Plant-based products made from vegetables, cereals, grains, and fruits have been shown to contain a large amount of natural antioxidants [4]. In addition to that, fruit and vegetable juices play an important role in delaying the onset of Alzheimer's disease especially among those who are at high risk for the disease [5]. The state of Sabah which is situated in Borneo Island has tropical rain forest is that contain a rich biodiversity of fruits and vegetables. One of the fruits which is still underutilized is *Cyphomandra betacea *that can grow naturally in the higher-humidity and low-temperature area.

**Figure 1:**
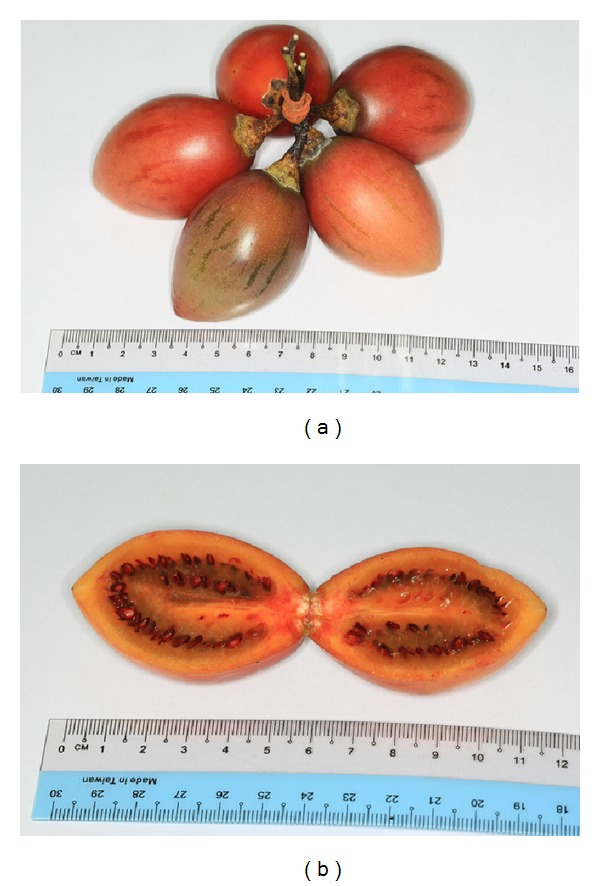
(a) Fruit of *C. betacea* and (b) Cross-section of *C. betacea*.

*C. betacea *is locally known as “Buah Cinta,” “Moginiwang,” or “Tamarillo” among local people in Sabah, Malaysia. Meanwhile, in Peninsular Malaysia, this plant is known as “Pokok Tomato” or “Tamarillo.” *C. betacea* is a semiwoody shrub with the tree up to 2-3 m high. The flowers are pink in colour. The fruit of *C. betacea* is reddish-brown to orange-red in colour depending on the fruit's maturity with the diameter of about 9–12 cm (Figures 1(a) and 1(b)). 87% of its total weight is made up by the flesh and seeds while the rest of its total weight is contributed by the skin of the fruit. The fruit is juicy and has smooth skin with egg-like shape. The fruit has like tomato-taste and ripe fruit of *C. betacea* is usually eaten raw by local community. This fruit can also be eaten with sugar to enhance the taste of the fruit. In addition to that, the fruit can also be processed to make juices, jams, jellies, desserts, and ice-cream toppings. This study was conducted to investigate the phytochemicals, antioxidant, and anticholinesterase potential of different parts of the fruit (i.e., skin and flesh).

## 2. Materials and Methods

### 2.1. Plant Materials and Sample Preparation

The fruit of *C. betacea* was collected from Penampang, Sabah, Malaysia, during October to December 2011. The herbarium specimens were identified and deposited in BORNEENSIS, Universiti Malaysia Sabah, Malaysia. The fruits were cleaned, weighed, and separated into flesh and peel. Small cut pieces were freeze-dried, and the freeze-dried samples were ground into fine powder using a dry grinder. The ground samples were sieved to get uniform size and then kept in an air-tight container and stored in a freezer (−20°C) until further analysis. 

### 2.2. Extraction

Samples (0.1 g) were extracted for 2 h with 80% methanol or distilled water with a ratio of 1 : 50 at room temperature on an orbital shaker set at 200 rpm [6]. The mixture was then centrifuged at 1400 ×g for 20 min and the supernatant was decanted into a 15 mL vial. The pellet was re-extracted under identical conditions. The supernatant was combined and used for total antioxidant activity, total phenolic, total flavonoid, and total anthocyanins contents, and total carotenoid content.

### 2.3. Determination of Total Phenolic Content

Total phenolic content was determined using Folin-Ciocalteu reagent as adapted from Velioglu et al. [6] with slight modifications. The extract (300 *μ*L) was mixed with 2.25 mL of Folin-Ciocalteu reagent (previously diluted 10-fold with distilled water) and allowed to stand at room temperature for 5 min. To this was added 2.25 mL of sodium carbonate (60 g/L) solution. After 90 min at room temperature, absorbance was measured at 725 nm using spectrophotometer. Standards of gallic acid in the concentration range 0 to 100 *μ*g/mL were run with the test samples, from which a standard curve was plotted. The result was expressed as mg gallic acid equivalents in 1 g of dried sample (mg GAE/g).

### 2.4. Determination of Total Flavonoid Content

Total flavonoid content was determined by using a colorimetric method described by Dewanto et al. [7] with slight modification. Briefly, 0.5 mL of extract was mixed with 2.25 mL of distilled water in a test tube followed by addition of 0.15 mL of 5% NaNO_2_ solution. 0.3 mL of a 10% AlCl_3_·6H_2_O solution was added after 6 min and allowed to stand for another 5 min before 1.0 mL of 1 M NaOH was added and allowed to stand for another 5 min. The mixture was mixed well with vortex and the absorbance measured immediately at 510 nm using spectrophotometer. Standards of rutin in the concentration range 0 to 100 *μ*g/mL were run with the test samples, from which a standard curve was plotted. The result were expressed as mg rutin equivalents in 1 g of dried sample (mg RE/g).

### 2.5. Determination of Total Anthocyanin Content

Total anthocyanin content was measured using a spectrophotometric pH differential protocol described by Giusti and Wrolstad [8] with slight modification. Briefly, 0.5 mL of the extract was mixed thoroughly with 3.5 mL 0.025 M potassium chloride buffer (pH 1). The mixture was mixed with vortex and allowed to stand for 15 min. The absorbance was then measured at 515 and 700 nm against a distilled water blank. The extract was then combined similarly with 0.025 M sodium acetate buffer (pH 4.5) and the absorbance was measured at the same wavelength after being allowed to stand for 15 min. The total anthocyanin content was calculated using the following equation:

(1) total  anthocyanin  content  (mg/100 g  of  dried  sample) =A×MW×DF×1000(ε×C),

where *A* is absorbance = (*A*_515_ − *A*_700_) pH 1.0 − (*A*_515_ − *A*_700_) pH 4.5, MW is the molecular weight for cyanidin-3-glucoside = 449.2; DF is the dilution factor of the samples, *ε* is the molar absorptivity of cyanidin-3-glucoside = 26900, and *C* is the concentration of the buffer in mg/mL. Results were expressed as mg of cyanidin-3-glucoside equivalents in 100 g of dried sample (mg c-3-gE/100 g dried sample).

### 2.6. Determination of Total Carotenoid Content

Total carotenoid content was measured by using the Hess et al. [9] method with slight modification. 300 *μ*L sample was added to 300 *μ*L distilled water and 600 *μ*L solvent. The mixture was mixed with 1.2 mL *n*-hexane. The mixture was centrifuged for 5 minutes using 4°C and reading was taken at 350 nm spectrophotometrically. Results were expressed as mg of *β*-carotene in 100 g of dried sample (mg BC/100 g dried sample).

### 2.7. DPPH Free Radical Scavenging Assay

The scavenging activity of the extract was measured by using 1,1-diphenyl-2-picrylhydrazyl DPPH as a free radical model and a method adapted from Magalhães et al. [10]. An aliquot (300 *μ*L) of samples or control (80% methanol or distilled water) was mixed with 3.0 mL of 500 *μ*M (DPPH) in absolute ethanol. The mixture was shaken vigorously and allowed to stand at room temperature for 30 min in the dark. Absorbance of the mixture was measured spectrophotometrically at 517 nm, and the free radical scavenging activity was calculated as follows:

(2) scavenging  effect  (%) =[1−{absorbance  of  sampleabsorbance  of  control}]×100.

The scavenging percentage of all samples was plotted. The final result was expressed as an EC_50_ value (the concentration of sample producing 50% scavenging of the DPPH radical; *μ*g/mL).

### 2.8. FRAP (Ferric Reducing/Antioxidant Power) Assay

This procedure was conducted according to Benzie and Strain [11] with slight modification. The working FRAP reagent was produced by mixing 300 mM acetate buffer (pH 3.6), 10 mM 2,4,6-tripyridyl-s-triazine (TPTZ) solution, and 20 mM FeCl_3_·6H_2_O in a 10 : 1 : 1 ratio prior to use and heated to 37°C in a water bath. A total of 3.0 mL FRAP reagent was added to a test tube and a blank reading was taken at 593 nm using a spectrophotometer. A total of 100 *μ*L of selected plant extracts and 300 *μ*L of distilled water were added to the cuvette. A second reading at 593 nm was performed after 90 min of incubation at 37°C in a water bath right after the addition of the sample to the FRAP reagent. The changes in absorbance after 90 min from the initial blank reading were compared with standard curve. Standards of known Fe (II) concentrations were run using several concentrations between 0 and 1000 *μ*g/mL. A standard curve was then plotted. The final result was expressed as the concentration of antioxidant having a ferric reducing ability in 1 gram of sample (*μ*M/g).

### 2.9. ABTS Decolorization Assay

The ABTS decolorization assay was carried out according to the method described by Re et al. [12] with slight modification. Working ABTS solution (7 mM) and potassium persulfate (2.45 mM) were added into a beaker and the mixture was allowed to stand 15 hours in the dark to generate an ABTS free radical cation solution. The mixture was diluted with 80% methanol or distilled water in order to obtain absorbance of 0.7 ± 0.2 units at 734 nm. To 2 mL of this working ABTS free radical cation solution, 200 *μ*L of methanolic or distilled water test solution was added, the mixture was vortexed for 45 seconds, and the resulting absorbance value read at 734 nm using microtiter plate reader. Standards of ascorbic acid in the concentration range 0 to 100 *μ*g/mL were run with the test samples, from which a standard curve was plotted. The final result was expressed as mg ascorbic acid equivalent antioxidant capacity in 1 g of sample (mg AEAC/g).

### 2.10. Anticholinesterase Inhibition Assay

The anti-cholinesterase inhibition assay was done according to Atta-ur-Rahman et al. [13]. 250 *μ*L phosphate buffer; 200 mM (pH 7.7) that contained fruit extracts was preincubated. 80 *μ*L of DTNB (3.96 mg of DTNB and 1.5 mg sodium bicarbonate dissolved in 10 mL phosphate buffer pH 7.7) and 10 *μ*L enzyme (2 U/mL) were added to the mixture. The mixture was incubated for 5 minutes at 25°C. 15 *μ*L of the substrate that contained 10.85 mg acetylthiocholine iodide in 5 mL phosphate buffer was added and incubated for 5 minutes. The colour developed was measured by using microwell plate reader at 412 nm. The percent of inhibition was calculated by using the following formula

(3) %  inhibition  =control  absorbance−sample/tested  absorbancecontrol  absorbance   ×100%.

## 3. Statistical Analyses

All experiments were carried out in 3 replicates in 3 independent experiments. The results were presented as mean ± standard deviation (SD) using Prism 5 software. The data were statistically analysed by one-way ANOVA and Duncan post hoc test. The level of statistical significance was set at *P* ≤ 0.05. Pearson's correlation analysis was done to correlate the phytochemicals and antioxidant potential in the samples.

## 4. Results and Discussion

### 4.1. Total Phenolic Content

The result of this study showed that the total phenolic content was higher in the peel as compared to the flesh for both 80% methanol and the aqueous extracts (*P* < 0.05) (Table 1). The results obtained for total phenolic in both peel and flesh extracts (Table 1) in the present study were similar to the study by Mertz et al. [14] on tree tomatoes which are estimated to have 308–570 mg phenolic content in 100 g of sample. A study by Ghosal and Mandal [15] on two Solanaceae fruits reported that the phenolic content in both *Solanum anguivi* and *Solanum incanum* is 1.61 and 2.31 mg/g, respectively, which is almost similar to the findings in this study. 

### 4.2. Total Flavonoid Content

The present study showed that the flesh and the peel of *C. betacea* displayed higher total flavonoid value than the flesh of the fruits. Total flavonoids content in *C. betacea* is suspected to have antilipid peroxidation effect because the previous study on tomatoes proved to have moderate anti-lipid peroxidation effect against goat liver due to the presence of polyphenols and flavonoids content [15]. Hence, the flavonoid content in these fruits suspected to contribute to the antioxidant in the fruits. 

### 4.3. Total Anthocyanin Content

The present study showed that anthocyanins were present in both the peel and flesh of the fruits, anthocyanins that give red, blue, pink, and purple colour of the plants [16] are suspected to give the red colour of *C. betacea* in this study. Total anthocyanin was higher in the flesh part as compared to the peel part. There is the significant difference in the total anthocyanins observed in both the peel and flesh part of *C. betacea* (*P* < 0.05) (Table 1).

The study by Mertz et al. [14] reported that pelargonidin rutinoside and delphinidin rutinoside were the two major anthocyanins in the red variety of the tree tomato. Delphinidin rutinoside, cyanidin rutinoside, and pelargonidin rutinoside have been identified previously as major anthocyanins in the tree tomato fruit from New Zealand [17]. These anthocyanins compounds are also suspected to be the major contributor of the anthocyanins in *C. betacea* in this study. 

### 4.4. Total Carotenoid Content

Rodriguez-Amaya et al. [18] reported that carotenoids were present in *C. betacea*. The present study showed that carotenoid content was higher in the flesh part of the fruits as compared to the peel (*P* < 0.05). Carotenoids have been known to have protective effects against degenerative or cardiovascular disease and to have the ability to act as antioxidants in some plants [19]. The study by Mertz et al. [14] confirmed that the major carotenoids content in unsaponified tree tomato is *β*-carotene while major carotenoids in saponified tree tomato are *β*-cryptoxanthin, lutein, and zeaxanthin. The antioxidant in the flesh part of *C. betacea* in this study is suspected to be contributed by the carotenoids content in the fruits. 

### 4.5. Scavenging Activity on 2,2-Diphenyl-2-picrylhydrazyl Radical

**Figure 2:**
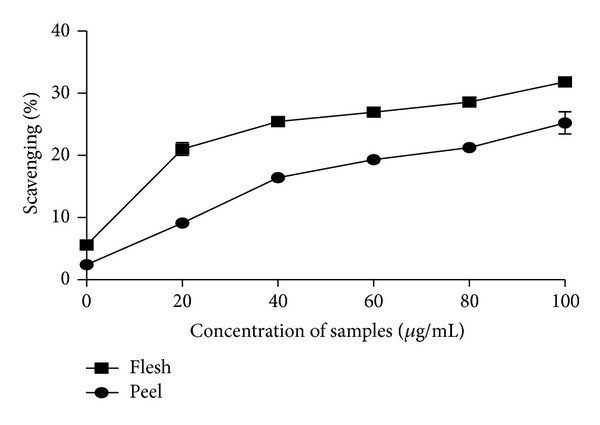
The scavenging activity of 80% methanol extract of different parts of *C. betacea* assayed by DPPH free radical scavenging method (*n* = 3).

**Figure 3:**
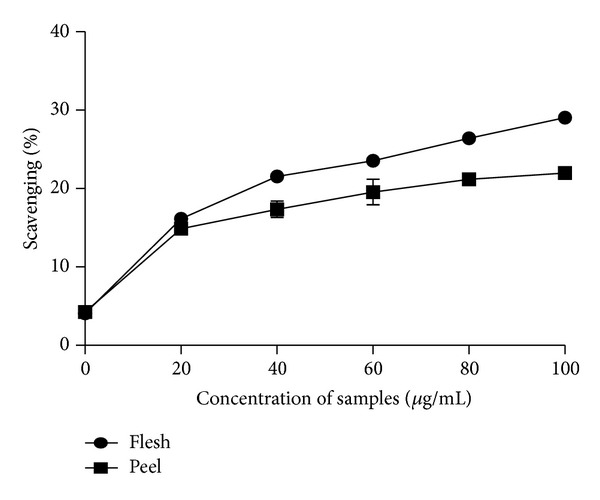
The scavenging activity of aqueous extract of different parts of *C. betacea* assayed by DPPH free radical scavenging method (*n* = 3).

For the 80% methanolic extracts, the flesh displayed higher scavenging effects as compared to the peel part of *C. betacea* (Figure 2). The scavenging activity of the aqueous extract displayed the same trend with higher scavenging activity observed in the flesh part followed by the peel of the fruit (Figure 3). 

 The flesh of *C. betacea* displayed higher antioxidant properties than the peel of *C. betacea* which is similar to a study by Azrina et al. [20] which found the highest scavenging activity displayed in the flesh part of *Canarium odontophyllum *as compared to the skin and kernel part of the fruits. *C. betacea* is rich in *β*-carotene and ascorbic acid which makes them good natural sources of provitamin A and vitamin C [21], and hence the *β*-carotene and ascorbic acid content in these fruits are suspected to contribute majorly to the antioxidant activity in the fruits.

### 4.6. Ferric Reduction Based on FRAP Assay

The reducing ability of the 80% methanol extracts of *C. betacea *was higher in the peel followed by the flesh. The same trend was also observed in the aqueous extract (Table 2). The FRAP value of the flesh *C. betacea* as a reductant in this study is almost similar to the cherry tomato as reported by George et al. [22], but the peel of *C. betacea *displayed higher reducing ability as compared to the cherry tomato.

Scalfi et al. [23] reported that small Corbarini tomatoes have high antioxidant activity. The peel of the tomatoes proved to have as abundant amount of lycopene which is suspected to contribute to the higher reducing capabilities in the peel of *C. betacea* in this study. Higher antioxidant properties in the nonedible part of *C. betacea* are in line with the study by Abu Bakar et al. [24] which showed higher reducing activity in the peel of the *Mangifera pajang *as compared to the flesh part of the fruit.

### 4.7. ABTS Scavenging Assay

For the 80% methanol extracts, the higher scavenging activity was found in the peel as compared to the flesh (Table 1). The result showed that the peel of *C. betacea *displayed higher antioxidant capacity as compared to the flesh of the fruit but not significantly different in the 80% methanolic extract (*P* > 0.05). Higher antioxidant activity in the peel of *C. betacea* is suspected to be contributed by the ascorbic acid and phenol in the peel of *C. betacea* [22]. The nonedible parts of *C. betacea* displayed higher antioxidant activity as compared to the edible parts of the fruits. The results of this study are in line with the study by Vasco et al. [25] which displayed higher antioxidant activity in the peel of Capuli cherry as compared to its pulp. 

### 4.8. Anticholinesterase Activity

**Figure 4:**
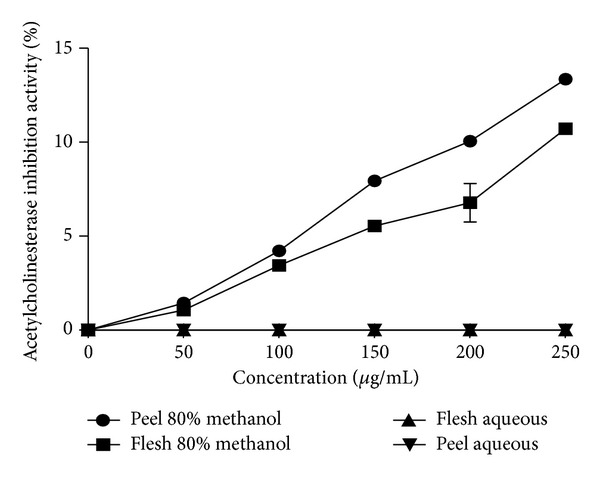
The anticholinesterase activity (%) of different parts of *C. betacea* for 80% methanol extract and aqueous extract (*n* = 3).

The present study showed that only the 80% methanolic extract of *C. betacea* has anti-cholinesterase activity while there was no anti-cholinesterase activity in the aqueous extract when tested at concentration of 50–250 *μ*g/mL (Figure 4). The peel of *C. betacea *displayed higher anti-cholinesterase activity as compared to the flesh part. The previous study on the fruits which come from the *Solanaceae* family showed that the fruits (edible part) of *Lycopersicon esculentum *Mill. displayed lower anti-cholinesterase activity as compared to the tuber part (nonedible part) of *Solanum tuberosum *L. (potato) which is in line with this study [26].

### 4.9. Relation between Phytochemicals, Antioxidants, and Anticholinesterase

The phenolics and flavonoids are phytochemicals that are suspected to contribute to the antioxidant activity in the peel of *C. betacea*. Meanwhile, anthocyanin and the carotenoids are two major components that might contribute to the antioxidant activity in the flesh parts of the fruits. Hence, the correlation analysis was performed to investigate the relationship between the phytochemicals and antioxidant activity in *C. betacea *extracts. 

The results showed that there is a strongly positive correlation between the FRAP and ABTS assays with the phenolic and flavonoids compound in the peel of *C. betacea*. FRAP assay displayed strong positive correlation with the phenolic and flavonoid content with the values of *r* = 0.880 and *r* = 0.843, respectively. ABTS assay showed strong positive correlation with total phenolic and flavonoid with the values of *r* = 0.812 and *r* = 0.898, respectively. 

The results of this study were in agreement with earlier literature by Ghosal and Mandal [15] which showed that the antioxidant activity in both *S. incanum* and *S. anguivi* was contributed mainly by the phenolic and flavonoids compound in the samples. Higher scavenging activity in the flesh has been shown to correlate with the presence of the anthocyanin and carotenoids compound in the flesh of the fruits with the values of *r* = 0.948 and *r* = 0.907, respectively. The results of this study are in line with a previous study by Kalt et al. [27] which proved that the antioxidant activity in the blueberries is strongly correlated with the anthocyanin content. A previous study showed that the carotenoid content in yellow and red tree tomato has significant effect on the antioxidant activity in that sample [14]. 

Meanwhile, the antioxidant activity also showed positive correlation with the anti-cholinesterase activity. However, the strength of correlation is based on the antioxidant assay used. The results showed that the anti-cholinesterase activity of the present study displayed weak, moderate, and strong positive correlation with DPPH, FRAP, and ABTS assays with the values of *r* = 0.199, *r* = 0.445, and *r* = 0.945, respectively. Previous study by Ferreira et al. [28] showed that most of the medicinal plants from Portugal have positive correlation between the antioxidant and acetylcholinesterase activity. 

From the results of this study, it can be concluded that *C. betacea* has a significant amount of phenolics, flavonoids, anthocyanin, and carotenoid which contribute to the antioxidant activity of the fruit extracts. The acceptable amount of phytochemicals in the fruits showed that *C. betacea* is one of the richest sources of antioxidant phytonutrients and has anti-cholinesterase properties that can enhance human health. 

## Figures and Tables

**Table 1:** Phytochemical contents of *C. betacea*  fruit extracts.

Samples	Total phenolics^1^	Total flavonoids^2^	Total anthocyanins^3^	Total carotenoid^4^
80% methanol				
Flesh	2.61 ± 0.12^b^	2.41 ± 0.02^b^	4.15 ± 0.04^a^	25.13 ± 0.35^a^
Peel	4.89 ± 0.04^a^	3.36 ± 0.01^a^	1.36 ± 0.10^c^	19.13 ± 1.93^b^
Aqueous				
Flesh	1.81 ± 0.02^d^	1.15 ± 0.01^c^	2.15 ± 0.14^b^	19.93 ± 0.05^b^
Peel	2.40 ± 0.01^c^	1.29 ± 0.02^b^	0.61 ± 0.03^d^	11.84 ± 0.63^c^

Values are presented as mean ± SD (*n* = 3) which, with different letters (within column), are significantly different at *P* < 0.05.

^1^Total phenolic content was expressed as mg gallic acid equivalents in 1 g of dried sample (mg GAE/g).

^2^Total flavonoid content was expressed as mg rutin equivalents in 1 g of dried sample (mg RE/g).

^3^Total anthocyanin content was expressed as mg of cyanidin-3-glucoside equivalents in 100 g of dried sample (mg C-3-GE/100 g dried sample).

^4^Total carotenoid content was expressed as mg of *β*-carotene in 100 g of dried sample (mg BC/100 g dried sample).

**Table 2:** Antioxidant properties of extracts of different parts of *C. betacea*, assessed by three different assays.

Samples	DPPH assay (%)^1^	FRAP assay^2^	ABTS assay^3^
80% methanol			
Flesh	31.82 ± 1.29^a^	1.95 ± 0.46^c^	39.14 ± 2.23^a^
Peel	23.94 ± 0.88^c^	9.33 ± 0.54^a^	40.14 ± 1.76^a^
Aqueous			
Flesh	29.04 ± 0.73^b^	0.67 ± 0.30^c^	19.71 ± 1.38^c^
Peel	21.76 ± 0.81^c^	6.04 ± 1.23^b^	27.18 ± 2.29^b^

Values are presented as mean ± SD (*n* = 3) which, with different letters (within column), are significantly different at *P* < 0.05.

^1^DPPH free radical scavenging activity was expressed as % of scavenging (*μ*g/mL).

^2^FRAP was expressed as mM ferric reduction to ferrous in 1 g of dry sample.

^3^ABTS free radical scavenging activity was expressed as mg ascorbic acid equivalent antioxidant capacity (AEAC) in 1 g of dry sample.
